# Analysis of differences in human leukocyte antigen between the two Wellcome Trust Case Control Consortium control datasets

**DOI:** 10.5808/GI.2019.17.3.e29

**Published:** 2019-09-27

**Authors:** Chloe Soohyun Jang, Wanson Choi, Seungho Cook, Buhm Han

**Affiliations:** Department of Biomedical Sciences, Seoul National University College of Medicine, Seoul 03080, Korea

**Keywords:** genome-wide association study, human leukocyte antigen, shared control

## Abstract

The Wellcome Trust Case Control Consortium (WTCCC) study was a large genome-wide association study that aimed to identify common variants associated with seven diseases. That study combined two control datasets (58C and UK Blood Services) as shared controls. Prior to using the combined controls, the WTCCC performed analyses to show that the genomic content of the control datasets was not significantly different. Recently, the analysis of human leukocyte antigen (HLA) genes has become prevalent due to the development of HLA imputation technology. In this project, we extended the between-control homogeneity analysis of the WTCCC to HLA. We imputed HLA information in the WTCCC control dataset and showed that the HLA content was not significantly different between the two control datasets, suggesting that the combined controls can be used as controls for HLA fine-mapping analysis based on HLA imputation.

## Introduction

Genome-wide association studies (GWAS) have identified many genetic variations influencing complex traits. However, the variants identified to date only explain a portion of heritability, possibly because there remain many unidentified variants with small effects [1,2]. To detect associations with small genetic effects, large study cohorts are required to obtain sufficient statistical power [3]. Therefore, there have been ongoing efforts to increase the size of study cohorts. One possible way to increase the sample size is to utilize publicly available cohorts. Publicly available control datasets can be particularly useful, because they can be used as controls for many different diseases.

One of the popular publicly available datasets is the Wellcome Trust Case Control Consortium (WTCCC) dataset [4]. The WTCCC study aimed to identify associations of genetic variations with seven diseases across the genome. The dataset consists of the genotype data of ~2,000 cases of each of the seven diseases and a common control set of ~3,000 individuals. The controls came from two sources: ~1,500 individuals from the 1958 British Birth Cohort (58C) and ~1,500 individuals selected from blood donors recruited as part of the WTCCC project (UK Blood Services [UKBS] controls). The WTCCC performed tests to check differences in the allele frequencies of genome-wide single-nucleotide polymorphisms (SNPs) between the 58C and UKBS groups. The results indicated that there were no significant differences between the two control groups despite subtle dissimilarities in the population groups sampled, DNA processing techniques, and age.

More recently, the analysis of human leukocyte antigen (HLA) genes has become widespread owing to the development of HLA imputation technology [5-7]. The HLA complex, which encodes the major histocompatibility complex (MHC) proteins and is located on chromosome 6 (Chr. 6p21.1-6p21.3), is the most polymorphic and genetically variable region in the human genome. HLA comprises two main groups; class I antigens (HLA-A, -B, and -C) and class II antigens (HLA-DR, -DQ, and -DP) which form key determinants of histocompatibility. Large-scale HLA association analyses were previously difficult due to the high cost of HLA typing, but have recently become more commonly feasible through the help of HLA imputation, which can predict HLA information from SNP data without HLA typing [6,8]. HLA association analysis is similar to other association analyses in that a large sample size is critical for the successful mapping of variants. Thus, the use of publicly available datasets such as the WTCCC control dataset can also be helpful in HLA analysis.

Here, we extend the between-control homogeneity analysis of the WTCCC study to HLA. Our goal was to systematically examine potential differences in HLA between the 58C and UKBS controls in the WTCCC dataset. Although the WTCCC project reported that there were only few differences in the genome-wide SNP frequencies between the two control groups, the difference in HLA region has not been examined before. If the difference in HLA is negligible between the two control groups, this might suggest that these combined controls can be used as additional controls for HLA fine-mapping association studies. For this purpose, we imputed HLA information for the two control datasets using recently developed HLA imputation technology [5] with a large European reference panel [9]. The imputation yielded information on HLA alleles, amino acid residues, and intragenic SNPs in binary markers. Then, we tested the significance of differences in these markers between the control datasets.

## Methods

### Summary of the cohorts

A detailed description of the study samples can be found in the original WTCCC GWAS paper [4]. In summary, the WTCCC dataset includes cases of seven major complex diseases: type 1 diabetes, type 2 diabetes, coronary heart disease, hypertension, bipolar disorder, rheumatoid arthritis and Crohn disease, each with ~2,000 individuals, and ~3,000 shared controls from two cohorts. Among them, we only used the two control cohorts. The control samples were derived from two sources: half from the 1958 Birth Cohort (58C) and the remainder from UKBS samples.

The 1958 Birth Cohort (also known as the National Child Development Study) consists of all births in England, Wales, and Scotland in 1958. The second control group consists of 1,500 blood donors recruited from the UKBS in England, Scotland, and Wales.

### SNP quality control and filtration

In the WTCCC dataset, 4,351 SNPs had already been excluded based on a Hardy-Weinberg equilibrium (HWE) test threshold of p < 5.7 × 10^-7^ in the combined set of 2,938 controls, as well as 93 SNPs with p-values < 5.7 × 10^-7^ from either a one- or two-degrees of freedom test for differences between the two control groups.

In this analysis, we only focused on autosomal variants (459,059 SNPs). We further performed additional SNP quality control (QC) ﬁlters. We removed SNPs that showed a genotype failure rate greater than 0.05, SNPs with a minor allele frequency (MAF) < 5%, and SNPs with an HWE p < 0.00001. After applying these QC criteria to the combined control cohort, we obtained 358,085 SNPs.

### Principal component analysis

We conducted principal component analysis (PCA) with PLINK (v1.90b) [10] (--pca) to analyze systematic differences in genome-wide allele frequencies between the two cohorts. PCA is a popular dimensionality reduction technique that can account for population stratification in genetic data [11]. Prior to applying PCA, we used PLINK to prune the SNP data in windows of 50 bps, removing a SNP from each pair of SNPs with an r^2^ > 0.2. A total of 71,083 SNPs remained after pruning. We extracted the top principal components (PCs) that explained the most variance in the genotype data. The top two and three PCs were used to build two- and three-dimensional PC plots. The top 20 components were used as covariates in the logistic regression for the genome-wide SNPs, as well as in the logistic regression for the HLA binary markers.

### Phase 2 HapMap dataset

We obtained the publicly available Phase 2 HapMap data for 270 individual samples from three different populations (Central European [CEU], Yoruba in Ibadan, Nigeria [YRI], and the East Asian combined sample of Japanese in Tokyo and Han Chinese in Beijing [Asian: JPT + CHB]) [12]. We used this dataset to perform PCA to quantify the population structure of the samples.

### Kolmogorov-Smirnov test

The Kolmogorov-Smirnov (K-S) test was used to assess the equality of frequency distributions between the 58C and UKBS cohorts. We calculated the corresponding D statistic and p-value of the test using the basic stats package in R. p-values were two-sided.

### Imputation of HLA alleles

We used SNP2HLA [5] to impute HLA information for the 58C and UKBS cohorts, where the HLA information was generated as binary markers for (1) HLA alleles, (2) amino acid residues, and (3) intragenic SNPs. For the reference panel for imputation, we employed the Type I Diabetes Genetics Consortium dataset, which consists of 5,225 individuals of European ancestry [9]. The genomic region was restricted to the MHC complex region (chr6:29-34Mb, hg18) on chromosome 6 before imputation. As a result, we obtained the imputed genotypes of 8,961 binary markers, which were composed of 424 markers for HLA alleles (e.g., HLA_A_0101; the presence of HLA-A*01:01 allele in the *HLA-A* gene), 1,276 markers for amino acid residues (e.g., AA_A_245_30020064_V; the presence of valine at the 245th amino acid position of the *HLA-A* gene), 1,390 markers for intragenic SNPs (e.g., SNP_A_30020064; the intragenic SNP at base position of 30020064 in the *HLA-A* gene), and 5,871 markers for genomic variants (e.g., rs969931).

### Allele frequency difference analysis between the two control cohorts

We performed association tests to compare allele frequencies between the control cohorts. We used the logistic regression model in PLINK v.1.90b (--logistic) [10]. To set the phenotypes, the UKBS cohort was set as “control” and the 58C cohort as “case.” We first conducted two-cohort association tests for genome-wide SNPs across the genome. Since we did not have regional information on the samples, which was used in similar analyses in the WTCCC study, we controlled for possible population stratification by including the top 20 PCs as additional covariates. For the HLA analysis, we tested all imputed markers, including HLA alleles, amino acids, and SNPs in the MHC region. Similar to the genome analysis, we included the same top 20 PCs as covariates in the HLA analysis.

## Results

### Principal component analysis

**Fig. 1.**
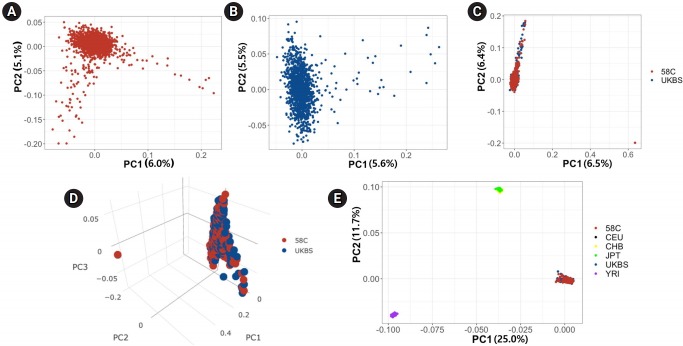
Principal component (PC) analysis of genotypic frequencies of genome-wide single-nucleotide polymorphism markers. (A) The first two PCs for 58C individuals. (B) The first two PCs for UK Blood Services (UKBS) individuals. (C) The first two PCs for 58C and UKBS individuals. (D) The first three PCs for 58C and UKBS individuals. (E) The first two PCs for 58C and UKBS individuals integrated with the HapMap populations (Phase2).

We first used PCA to analyze differences between the two control cohorts at the population level. We calculated PCs in four ways: (1) using the 58C cohort only, (2) using the UKBS cohort only, (3) using the two cohorts combined, and (4) using the combined set with the HapMap data (N=270) integrated. We applied SNP QC filters before PCA (see Methods). PCs were calculated based on the variance-standardized relationship matrix among the individuals included in each analysis. Fig. 1A and 1B show the PCs in the 58C and UKBS cohorts, respectively, and Fig. 1C shows the results when the two cohorts were merged. Fig. 1C demonstrates high similarity in the ranges of the first two PCs between the two cohorts, except for two outliers (one in each cohort) at the bottom right corner of the plot. Fig. 1D is a tridimensional plot of the PCs of the two control cohorts. The red sphere corresponds to the 58C group, and the blue indicates the UKBS group. In the 3-PC plot, similar to the 2-PC plot, the two cohorts were not visually distinct from each other. Fig. 1E presents the PCs of the WTCCC merged with three HapMap populations (CEU, YRI, and Asian [CHB, JPT]). The plot shows that the 58C, UKBS, and CEU cohorts were grouped together in the PC plot, showing that the two control cohorts had European ancestry homogeneous to the CEU.

### SNP allele frequency distribution of the two cohorts

**Fig. 2.**
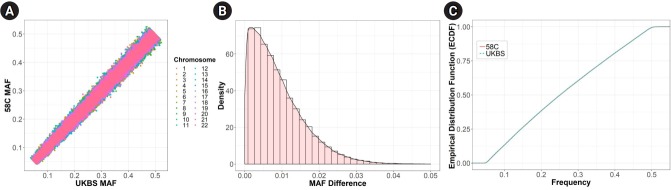
Comparison of single-nucleotide polymorphism minor allele frequency (MAF) between the two cohorts. (A) MAF comparison of the two cohorts. (B) Density plot of the absolute MAF difference of the two cohorts. (C) ECDF curve plot for the Kolmogorov-Smirnov test. UKBS, UK Blood Services.

We examined the allele frequencies of genome-wide SNPs in the two cohorts, using 358,085 autosomal SNPs from 2,938 samples (total genotyping rate, 0.996). Table 1 provides descriptive statistics of the allele frequency distribution in each cohort. These statistics did not show any distinctive differences between the two cohorts. In addition, Fig. 2A and 2B show the patterns of the MAF differences. We performed the K-S test, a general nonparametric method for comparing the density distributions of two datasets. Fig. 2C shows a plot of the empirical cumulative density function (ECDF) curves of the two cohorts. The K-S test returns a D statistic, which refers to the absolute maximum distance between the ECDF of the two samples and a p-value corresponding to the D statistic. D was 0.015 and the p-value was 0.819; therefore, we could not reject the null hypothesis that the two datasets were drawn from the same distribution.

### Allele frequency association test between the two cohorts

Next, we performed a GWAS to detect genomic variants that showed significant differences in the MAF between the two cohorts. In the original study, the WTCCC performed a similar test to check differences in the allele frequencies between the 58C and UKBS cohorts using genome-wide scans. In that analysis, they stratified the samples based on their broad geographical origin (12 UK regions). The result of the test statistics in a quantile-quantile (Q-Q) plot indicated that there were no significant differences between the two control groups. They showed that their samples did not show a clear population structure in multidimensional scaling and PCA plots, despite the 12 different origins of the samples.

Our association analysis was similar to the analysis in the original paper, but different in that we included the top 20 PCs as covariates instead of performing a stratified analysis based on 12 geographical regions. The reason for this difference is that the geographical origin information was not readily available for download from the WTCCC website. To our knowledge, only the genotype data and the disease status were publicly available to researchers. Thus, in general, a researcher using the WTCCC controls would likely use only PCs as covariates instead of geographical information. Moreover, even if a researcher were to obtain the geographical region information for the WTCCC controls, it would not possible to use this information as a covariate in the case/control analysis without having the same information for cases. Therefore, it is worthwhile to examine the differences between the two control cohorts in an analysis using only PCs as covariates.

**Fig. 3.**
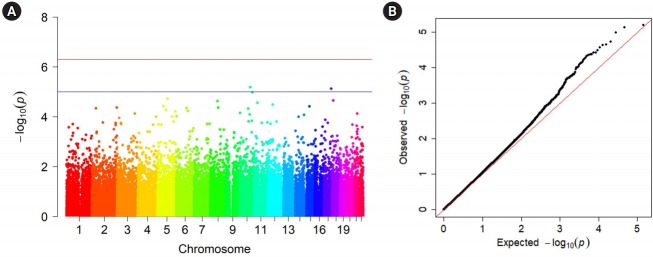
Genome-wide association results and quantile-quantile (Q-Q) plots for genome-wide association study p-values. (A) Manhattan plot of genome-wide association results. Within each chromosome position shown on the x axis, log10 (p-values) of the association statistics are visualized on the y-axis. The red line denotes genome-wide significance (5.7 × 10^-7^). (B) Q-Q plot of association p-values. Red line denotes the expected values under no inflation.

Specifically, we tested the MAF difference between the two cohorts using logistic regression, while including the top 20 PCs as covariates to account for regional differences in the samples. To this end, we set one cohort as cases (58C) and another cohort as controls (UKBS). We then performed association tests to identify the associations between SNPs and the cohort identifier. Fig. 3A shows the Manhattan plot of associations. We did not find any loci that exceeded the threshold for genome-wide significance of 5.7 × 10^-7^ (red line), which was the threshold used in the WTCCC study. This is unsurprising because the dataset we used was already filtered to remove any SNPs with p < 5.7 × 10^-7^ in the MAF difference test of the original study. However, we could find several loci with moderate significance (p < 1 × 10^-5^, blue line). Fig. 3B demonstrates that the association tests had slight inflation (genomic control inflation factor [λ]: 1.04). Upon visual inspection, the Q-Q plot appeared slightly more inflated in our analysis than the Q-Q plot presented in the original paper (Fig. 3B of the WTCCC study [4]), possibly because the exact regional information was not included in the analysis. An exact comparison of inflation was not possible because the result of the inflation statistic in the original study was not available.

### HLA allele frequency distribution of the two cohorts

**Fig. 4.**
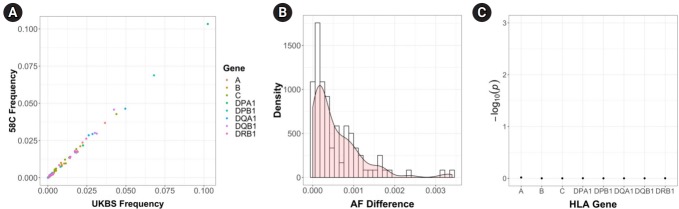
Comparison of human leukocyte antigen (HLA) allele frequency between the two cohorts. (A) HLA allele frequency comparison of the two cohorts. (B) Density plot of the absolute HLA allele frequency difference of the two cohorts. (C) p-value of the Kolmogorov-Smirnov test in each of the HLA genes. UKBS, UK Blood Services; AF, allele frequencies.

Next, we examined the HLA allele frequencies in each cohort. We imputed the HLA information using SNP2HLA. The imputation gave us binary markers for (1) HLA alleles, (2) amino acid residues, and (3) intragenic SNPs. Using imputed binary markers, we extracted two-digit and four-digit HLA alleles for each sample. As a result, we obtained a total of 23,504 imputed alleles for eight genes in 2,938 samples. We focused on the two-digit alleles in this analysis. When we counted the number of unique two-digit alleles in each gene, we found 18 alleles for *HLA-A*, 27 alleles for *HLA-B*, 13 alleles for *HLA-C*, three alleles for *HLA-DPA1*, 17 alleles for *HLA-DPB1*, six alleles for *HLA-DQA1*, five alleles for *HLA-DQB1*, and 13 alleles for *HLA-DRB1*. Most of the alleles (97) were observed in both cohorts, and five alleles were detected in a single cohort (two alleles for 58C and three alleles for UKBS). The two-digit HLA allele frequencies are listed in Supplementary Table 1. Table 2 provides the descriptive statistics of the two-digit HLA allele frequency distribution in each cohort. These statistics did not show distinctive differences between the two cohorts. The mean absolute difference in the HLA allele frequencies was 0.00562. Fig. 4A and 4B show the patterns of the HLA allele frequency differences. We then performed the K-S test for each HLA gene, and the results are presented in Fig. 4C. All the HLA genes showed non-significant p-values in the K-S test, demonstrating that there was no significant difference in the imputed HLA allele frequencies between the two control cohorts.

### HLA association test between the two cohorts

**Fig. 5.**
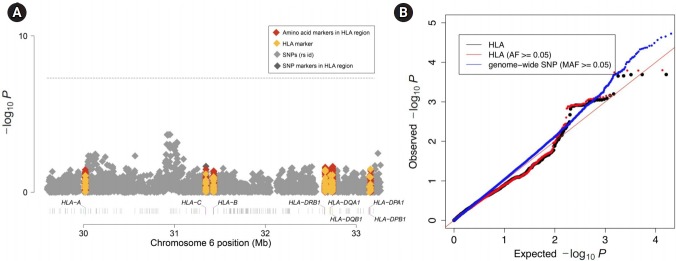
Imputed human leukocyte antigen (HLA)-wide association results and quantile-quantile (Q-Q) plots for p-values. (A) Manhattan plot of MHC complex region-wide association results. The p-values are from the binary markers for imputed HLA alleles (yellow), amino acid residues (red), genotyped single-nucleotide polymorphisms (SNPs) (gray) and intragenic SNPs (deep gray). (B) Q-Q plots of association p-values for MHC binary markers (black), MHC binary markers with allele frequencies (AF)≥0.05 (red), and genome-wide SNPs (blue). Red line denotes the expected values under no inflation. MAF, minor allele frequency.

Finally, we tested the HLA difference between the two cohorts using logistic regression. Similar to the genome-wide analysis above, we included the top 20 PCs as covariates to account for regional differences among the samples. Again, we set one cohort as cases (58C) and the other cohort as controls (UKBS). We then performed association tests to identify associations between markers in the MHC region (including both the imputed binary markers and the genotyped SNPs) and the cohort identifier. Fig. 5A shows the Manhattan plot of associations. We did not find any binary marker that exceeded the threshold for genome-wide significance of 5.7 × 10^-7^ (red line), which was the threshold used in the WTCCC study. Fig. 5B demonstrates that the association tests did not show inflation (λ = 0.92 for all markers and λ = 0.93 for markers with a frequency ≥ 0.05). Overall, both the Manhattan plot and the Q-Q plot showed that there was no significant difference in HLA between the two control cohorts, emphasizing that the two control cohorts can be used as a single combined control for HLA analyses.

## Discussion

In the present study, we compared the two control cohorts of the WTCCC dataset (UKBS and 58C). We focused on between-cohort differences in the HLA genes, which belong to the polymorphic region of chromosome 6. In both the association analysis of the genome-wide SNPs and the association analysis of the imputed HLA markers, no alleles showed a significant difference in allele frequency between the cohorts. Furthermore, in the K-S test, the two cohorts did not show statistically significant differences in any HLA gene. These results suggest that the two control cohorts are genetically homogeneous in HLA, and that the combined controls of the WTCCC can be used as supplementary controls for HLA fine-mapping studies as well, as for GWAS. However, we note that researchers must evaluate homogeneity between their own control cohort and the combined WTCCC controls if they are to use the WTCCC data as additional controls, because we only evaluated the homogeneity between the two WTCCC control datasets themselves.

## Figures and Tables

**Table 1.** Summary statistics of MAF for single-nucleotide polymorphisms

	UKBS MAF	58C MAF	Absolute MAF difference
Min	0.05	0.05	0
1st quantile	0.145	0.1448	0.0033
Median	0.2507	0.251	0.0072
3rd quantile	0.3709	0.3709	0.0125
Max	0.5	0.5	0.0616
Mean	0.2592	0.2597	0.0008

MAF, minor allele frequency; UKBS, UK Blood Services.

**Table 2.** Summary statistics of HLA AF

	UKBS AF	58C AF	Absolute AF difference
Min	0	0	0
1st quantile	0.001	0.001	0.0001
Median	0.0036	0.0033	0.0003
3rd quantile	0.0141	0.0139	0.0008
Max	0.1025	0.1033	0.0034
Mean	0.0098	0.0098	0.0005

HLA, human leukocyte antigen; AF, allele frequencies; UKBS, UK Blood Services.

## Supplementary Materials

Supplementary Table 1. Lists of 2-digit HLA allele frequencies
