# Supplementary material for: Analysis of differences in human leukocyte antigen between the two Wellcome Trust Case Control Consortium control datasets
Source: Genomics Inform. 2019 Sep 27;17(3):e29. doi: 10.5808/GI.2019.17.3.e29 (PMC6808636; doi:10.5808/GI.2019.17.3.e29)
Supplement: Supplementary Table 1. — Lists of 2-digit HLA allele frequencies [file gi-2019-17-3-e29-suppl.pdf]

Supplementary Table 1. Lists of 2-digit HLA allele frequencies

Allele	58C AF	NBS AF
HLA-A*01	0.02362	0.02221
HLA-A*02	0.03685	0.03662
HLA-A*03	0.01754	0.01852
HLA-A*11	0.00735	0.00802
HLA-A*23	0.00194	0.0024
HLA-A*24	0.00955	0.01042
HLA-A*25	0.00161	0.00184
HLA-A*26	0.00321	0.00382
HLA-A*29	0.00554	0.00433
HLA-A*30	0.0027	0.0027
HLA-A*31	0.0033	0.0027
HLA-A*32	0.00507	0.00493
HLA-A*33	0.0008	0.00111
HLA-A*34	0.00013	0.00026
HLA-A*36	0	0.00004
HLA-A*66	0.00038	0.0006
HLA-A*68	0.00532	0.00442
HLA-A*69	0	0.00009
HLA-B*07	0.01741	0.01912
HLA-B*08	0.01758	0.01724
HLA-B*13	0.00211	0.0024
HLA-B*14	0.00494	0.00506
HLA-B*15	0.01001	0.0084
HLA-B*18	0.00477	0.00527
HLA-B*27	0.00617	0.00527
HLA-B*35	0.00782	0.00888
HLA-B*37	0.00207	0.00189
HLA-B*38	0.00063	0.00099
HLA-B*39	0.0019	0.0018
HLA-B*40	0.00866	0.00888
HLA-B*41	0.00063	0.00047
HLA-B*44	0.02113	0.02075
HLA-B*45	0.00106	0.00099
HLA-B*46	0.00017	0.00004
HLA-B*47	0.00042	0.00026
HLA-B*49	0.00152	0.00154
HLA-B*50	0.00127	0.0015
HLA-B*51	0.00461	0.00433
HLA-B*52	0.00089	0.0006
HLA-B*53	0.00021	0.00013
HLA-B*55	0.00232	0.00274
HLA-B*56	0.00046	0.00051

HLA-B*57	0.00549	0.00472
HLA-B*58	0.00063	0.00103
HLA-B*73	0	0.00004
HLA-C*01	0.00482	0.00489
HLA-C*02	0.00423	0.0042
HLA-C*03	0.01927	0.01822
HLA-C*04	0.00963	0.01145
HLA-C*05	0.01356	0.01364
HLA-C*06	0.01217	0.01098
HLA-C*07	0.04276	0.04391
HLA-C*08	0.00494	0.00493
HLA-C*12	0.00423	0.00424
HLA-C*14	0.00101	0.0009
HLA-C*15	0.00194	0.00193
HLA-C*16	0.00596	0.00506
HLA-C*17	0.00055	0.00069
HLA-DPA1*01	0.10331	0.10248
HLA-DPA1*02	0.02172	0.02251
HLA-DPA1*03	0.00004	0.00004
HLA-DPB1*01	0.00744	0.00823
HLA-DPB1*02	0.01711	0.01745
HLA-DPB1*03	0.01399	0.01411
HLA-DPB1*04	0.06879	0.068
HLA-DPB1*05	0.00211	0.00304
HLA-DPB1*06	0.00215	0.00163
HLA-DPB1*09	0.00076	0.0009
HLA-DPB1*10	0.00241	0.00206
HLA-DPB1*11	0.00321	0.00296
HLA-DPB1*13	0.00161	0.00219
HLA-DPB1*14	0.00144	0.00137
HLA-DPB1*15	0.00085	0.00094
HLA-DPB1*16	0.00093	0.00086
HLA-DPB1*17	0.00173	0.00103
HLA-DPB1*19	0.00025	0.00026
HLA-DPB1*20	0.00017	0
HLA-DPB1*23	0.00004	0
HLA-DQA1*01	0.0464	0.04961
HLA-DQA1*02	0.018	0.01737
HLA-DQA1*03	0.02852	0.02616
HLA-DQA1*04	0.00262	0.00296
HLA-DQA1*05	0.02937	0.02856
HLA-DQA1*06	0.00008	0.00039
HLA-DQB1*02	0.03013	0.0301

HLA-DQB1*03	0.04576	0.04236
HLA-DQB1*04	0.0027	0.00296
HLA-DQB1*05	0.01682	0.01831
HLA-DQB1*06	0.02966	0.0313
HLA-DRB1*01	0.01407	0.01445
HLA-DRB1*03	0.01758	0.01732
HLA-DRB1*04	0.02624	0.02461
HLA-DRB1*07	0.01796	0.01737
HLA-DRB1*08	0.0027	0.00334
HLA-DRB1*09	0.00186	0.00124
HLA-DRB1*10	0.00025	0.00039
HLA-DRB1*11	0.00849	0.0072
HLA-DRB1*12	0.00182	0.00219
HLA-DRB1*13	0.01323	0.01411
HLA-DRB1*14	0.00224	0.00334
HLA-DRB1*15	0.01783	0.01887
HLA-DRB1*16	0.00072	0.00051
